# Acid-sensing ion channel 2a assembles with epithelial Na^+^ channel β and γ subunits to form mechanosensitive ion channels

**DOI:** 10.1016/j.jbc.2026.113151

**Published:** 2026-05-14

**Authors:** Shujie Shi, Sarah Christine M. Whelan, Kennedy G. Szekely, Ossama B. Kashlan, Heather Drummond, Thomas R. Kleyman

**Affiliations:** 1 Renal-Electrolyte Division, Department of Medicine, University of Pittsburgh, Pittsburgh, Pennsylvania, USA; 2 Department of Computational and Systems Biology, University of Pittsburgh, Pittsburgh, Pennsylvania, USA; 3 Physiology and Biophysics, University of Mississippi Medical Center, Jackson, Mississippi, USA; 4 Department of Cell Biology, University of Pittsburgh, Pittsburgh, Pennsylvania, USA; 5 Department of Pharmacology and Chemical Biology, University of Pittsburgh, Pittsburgh, Pennsylvania, USA

**Keywords:** ASIC, ENaC, mechanotransduction, oocytes, shear stress

## Abstract

ASIC2 and ENaC subunits are required for stretch-induced mechanoreceptor currents in renal vascular smooth muscle cells and pressure-induced constriction responses in small renal arteries and arterioles. To examine whether ENaC subunits and ASIC2 can form mechanosensitive ion channels, we co-expressed ASIC2a with ENaC β and γ subunits in *Xenopus* oocytes to test the channel’s response to flow-induced shear stress by changing the perfusion rates with Na^+^ as the conducting ion. Under baseline conditions (0.5 ml/min), oocytes co-expressing ASIC2a and β/γENaC (ASIC2a+β/γENaC) displayed larger inward Na^+^ currents than ASIC2a alone. In oocytes expressing ASIC2a+β/γENaC, but not ASIC2a alone, baseline currents increased in response to a fast perfusion (5 ml/min). The inward Na^+^ currents carried by ASIC2a+β/γENaC were insensitive to 10 μM amiloride but were inhibited when extracellular Na^+^ was replaced with NMDG^+^. A gain-of-function ASIC2a mutant, ASIC2aG430V, had readily detectable amiloride-sensitive baseline currents. Interestingly, flow-mediated channel activation was dependent on subunit composition. A high perfusion rate (5 ml/min) elicited a two-fold increase in Na^+^ currents in oocytes expressing ASIC2aG430V or ASIC2aG430V+βENaC, which was further increased to approximately four-fold when γENaC or both β and γ subunits were co-expressed with ASIC2aG430V (ASIC2aG430V+γENaC or ASIC2aG430V+β/γENaC). Exposure to acidic pH evoked greater channel activity in oocytes expressing ASIC2aG430V+β/γENaC than ASIC2aG430V alone. In contrast, ENaC β and γ subunits did not alter the channel’s permeability to monovalent cations of different sizes. Collectively, our experimental findings and structural modeling suggest that ASIC2a assembles with ENaC β and γ subunits to form mechanosensitive channels.

The epithelial sodium channel (ENaC)/Degenerin family encodes a group of structurally related ion channels participating in mechanosensing and signal transduction (1, 2, 3). *Caenorhabditis elegans* degenerins are expressed in touch receptor neurons and initiate the gentle touch response (4, 5). *Drosophila* pickpockets are expressed in sensory neurons and are required for larval locomotion (6, 7, 8, 9). The mammalian acid-sensing ion channels (ASICs) participate in mechanosensation and nociception in both the central and peripheral nervous systems (10, 11). ENaC mediates the rate-limiting step of Na^+^ uptake in the distal nephron, and this process is regulated by fluid shear force (12, 13, 14, 15, 16).

Pressure-induced vasoconstriction is initiated by intraluminal pressure-induced stretch of vascular smooth muscle cells (SMCs) in small arteries and arterioles in most organs (17, 18). The specific mechanism(s) responsible for the initial mechano-electrical transduction event remain unresolved. We proposed that members of the ENaC/Degenerin family mediate the stretch-induced mechanoreceptor currents in renal vascular SMCs, as stretched-induced mechanoreceptor currents in these cells were largely inhibited in βENaC^m/m^ mice (19). We previously showed that ENaC subunits and ASIC2 are expressed in SMCs where they play essential roles in pressure-induced constriction (20, 21, 22, 23, 24, 25). Myogenic constriction is impaired in renal afferent arterioles isolated from mice with reduced βENaC expression (βENaC^m/m^) or ASIC2 null mice (26, 27).

The resolved structures of ASICs and ENaCs revealed that the channel assembles as a homo- or heterotrimer with the pore formed by the two transmembrane helices from each subunit, and with a highly organized extracellular domain (28, 29, 30, 31, 32, 33). Canonical ENaCs are a heterotrimer of αβγ or δβγ (33, 34, 35), while ASIC subunits (1a, 2a, and 3) are known to form homo- or heterotrimeric channels (36, 37, 38, 39). While animal models have not provided direct evidence of the assembly of specific ENaC and ASIC subunits, there is growing evidence that ENaC subunits hetero-oligomerize with ASIC1a to form functional channel complexes in native cells or heterologous expression systems (40, 41, 42, 43). When expressed in D54-MG glioma cells, ASIC1 forms a complex with ENaC α and γ subunits, which migrates at approximately 480 kDa in high-resolution, clear native gels (43). When co-expressed with ENaC α, β, or δ subunits in oocytes, ASIC1 became significantly less permeable to K^+^ and more sensitive to *P. cambridgei* venom (42). Single-channel recordings from alveolar epithelial cells in which either αENaC or ASIC1a were knocked down revealed the presence of two channel types: a highly cation-selective channel that contains the three ENaC subunits, and non-cation-selective channels that contain ASIC1a and αENaC (40). Furthermore, alveolar fluid clearance was reduced in ASIC1a knockout mice, which was further blunted by amiloride treatment (40).

We have shown that βENaC interacts with ASIC2 in vascular SMCs to mediate pressure-induced constriction in renal afferent arterioles (44). However, it is still unclear whether ASIC2 and ENaC subunits form functional channels that are gated by mechanical forces. In the current study, we expressed either the wild-type ASIC2a or a degenerin mutant, ASIC2aG430V, in *Xenopus* oocytes and examined whether the presence of ENaC β and/or γ subunits alters the channel’s response to flow-mediated shear stress. Our results suggest that ASIC2a interacts with both ENaC β and γ subunits to form mechanosensitive cation channels.

## Results

### Hybrid channels consist of mouse ASIC2a and ENaC β and γ subunits are activated by flow-mediated shear stress

**Figure 1:**
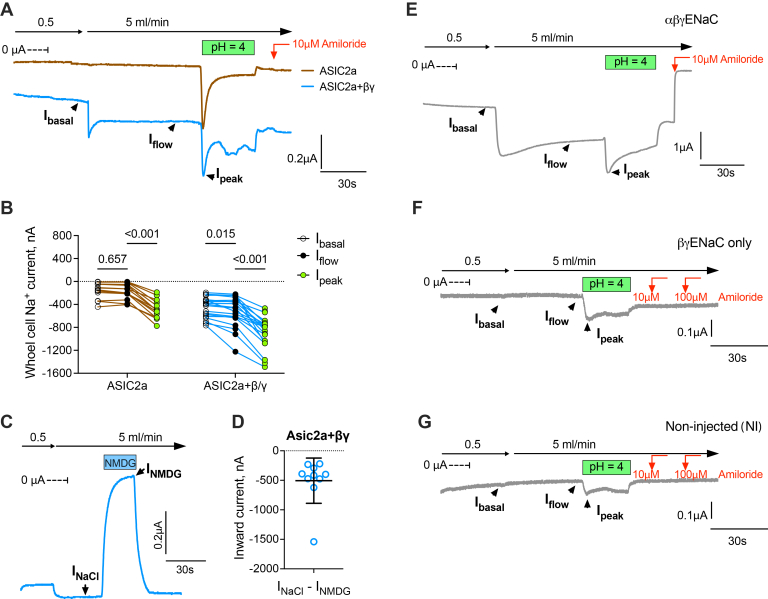
**Channels formed by ASIC2a and β and γENaC subunits are activated by flow-mediated shear stress.***A*, representative current traces of oocytes expressing wild type (WT) mouse ASIC2a or ASIC2a with mouse β and γENaC. Oocytes were initially perfused with a 110 mM NaCl buffer (NaCl-110, pH = 8) at a flow rate of 0.5 ml/min. The effect of shear stress on channel activity was measured by transitioning to a 10-fold higher flow rate (5 ml/min). The expression of ASIC2a was confirmed by switching to an acidic buffer (pH = 4). At the end of the experiment, 10 μM amiloride was added to the bath. *B*, summary of flow activation and pH response (I_basal_, I_flow_ and I_peak_) in oocytes expressing ASIC2a (n = 14) or ASIC2a+βγENaC (n = 19). Statistical comparisons were performed with a 2-way ANOVA, followed by Tukey's multiple comparisons test. *C*, representative current trace of oocytes expressing mouse ASIC2a+βγENaC at a low and high flow rate. Inward currents were not detected when bath Na^+^ was replaced with NMDG. *D*, summary of inward currents (I_NaCl_-I_NMDG_) in ten oocytes expressing ASIC2a+βγENaC. The effect of flow-mediated shear stress and acidic pH were also examined in oocytes expressing mouse αβγENaC (*E*), βγENaC alone (*F*), or non-injected control oocytes (*G*).

*Xenopus* oocytes were injected with cRNAs encoding WT mouse ASIC2a alone or with ENaC β and γ subunits, and the channel’s mechanosensitivity was assessed by increasing the perfusion rates from 0.5 to 5 ml/min. We also tested the channel’s response to changes in pH (from pH = 8 to pH = 4) with Na^+^ as the major conducting ion and the response to 10 μM amiloride (Fig 1*A*). When perfused at the slow rate (baseline), we detected larger inward Na^+^ currents in oocytes co-expressing ASIC2a with ENaC β and γ subunits (ASIC2a + β/γENaC) than ASIC2a alone (−447.3 ± 42.2 pA *vs* −152.2 ± 36.8 pA, *p* < 0.001). More importantly, only channels containing both ASIC2a and ENaC β and γ subunits exhibited shear stress-mediated channel activation. In oocytes expressing ASIC2a + β/γENaC, but not ASIC2a alone, baseline currents increased in response to a fast perfusion rate (5 ml/min), which were further activated by acidic pH (Fig. 1*B*), suggesting the hybrid channel contains properties of both ASIC and ENaC. The inward currents carried by ASIC2a + βγENaC were insensitive to 10 μM amiloride (Fig. 1*A*), a concentration that blocks ENaC currents (Fig. 1*E*) but were inhibited when extracellular Na^+^ was replaced with the large, impermeant cation NMDG^+^ (Fig. 1, *C* and *D*). As expected, amiloride-sensitive Na^+^ currents were activated by the high flow rate (5 ml/min) in oocytes expressing mouse αβγENaC (Fig. 1*E*). In contrast, we were not able to detect amiloride-sensitive Na^+^ currents in oocytes expressing only βγENaC (Fig. 1*F*) or in non-injected oocytes (Fig. 1*G*) at baseline or under a high perfusion rate (5 ml/min). Acidic pH elicited a small change in channel activity in oocytes expressing βγENaC (136 ± 84 nA, n = 9), which is indistinguishable to that in non-injected oocytes (186 ± 105 nA, n = 8, *p* = 0.30), suggesting that oocytes have endogenous acid activated channels. Collectively, our findings suggest that ASIC2a assembles with ENaC β/γ subunits to form mechanosensitive channels.

### A degenerin mutation of ASIC2a (G430V) retains its gating properties

**Figure 2:**
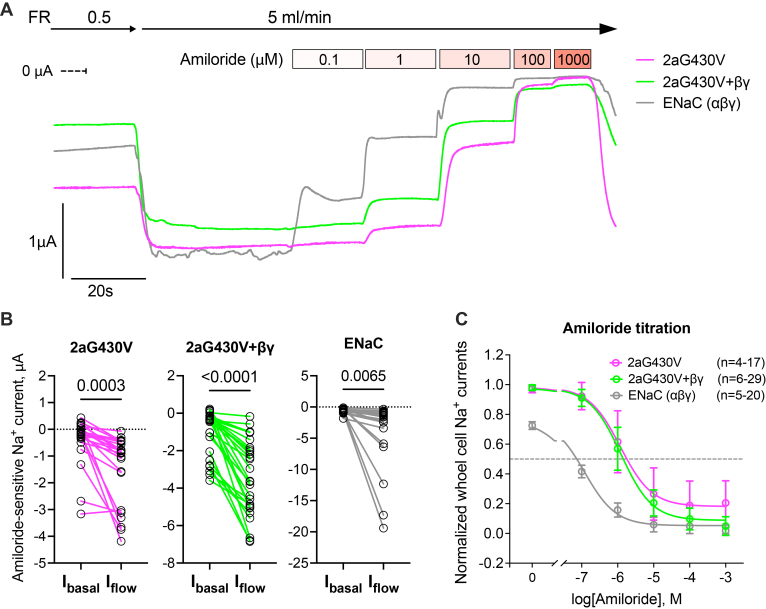
**Channels with a gain-of-function mutation in ASIC2a (G430V) and β and γENaC are activated by flow-mediated shear stress.***A*, representative current traces of oocytes expressing mouse αβγENaC, ASIC2aG430V or ASIC2aG430V+βγENaC. The effect of shear force on channel activity was measured by changing the perfusion rate from 0.5 ml/min to 5 ml/min in the NaCl-110 buffer. The channel’s sensitivity to amiloride was tested with increasing concentrations of amiloride (0.1, 1, 10, 100 or 1000 μM). *B*, summary of baseline currents (I_basal_) and flow-activated currents (I_flow_) of oocytes expressing αβγENaC (n = 29), ASIC2aG430V alone (n = 22) or ASIC2aG430V and βγENaC (n = 27). Statistical comparisons were performed with a paired Student’s *t* test. *C*, summary of current inhibition by amiloride. Whole cell Na ^+^currents measured in the presence of each amiloride concentration were normalized to the I_flow_ before amiloride application and fit to the dose-response equation described under “Experimental Procedures”. The number of oocytes (n) assayed for each group is indicated. Data were presented as mean ± SD. Statistical comparisons were performed with 2-way ANOVA, followed by Tukey's multiple comparisons test.

The baseline currents in oocytes expressing wild-type (WT) ASIC2a are typically very small, even after a prolonged incubation (>72 h) following a cRNA injection (Fig. 1*A*) that leads to a decline in oocyte survival and recording quality. To address this issue, we introduced a gain-of-function mutation at the degenerin site of ASIC2a, namely, ASIC2aG430V. It has been previously shown that introducing bulky residues at the degenerin site in ASIC2 drastically increased the channel’s sensitivity to amiloride without modification of its conductance or ion selectivity (45). Oocytes injected with WT αβγENaC were included as a control. 48h following cRNA injection, inward Na^+^ currents were readily detected in oocytes expressing ASIC2aG430V or ASIC2aG430V+βγENaC, which is comparable to that of αβγENaC under the low flow rate. The baseline currents were further augmented by a higher flow rate in oocytes expressing ASIC2aG430V+βγENaC or ASIC2aG430V alone (Fig. 2, *A* and *B*). We also examined the channel’s sensitivity to amiloride (Fig. 2*A*). Na^+^ currents in oocytes expressing ASIC2aG430V or ASIC2aG430V+βγENaC were gradually blocked by increasing concentrations of amiloride to a similar extent, with an IC_50_ of approximately 1.2 μM, 10-fold higher than the IC_50_ observed with αβγENaC (Fig. 2*C*). Overall, the gain-of-function mutant, ASIC2aG430V had higher baseline activity and amiloride sensitivity than the WT ASIC2a and exhibited a response to flow-induced shear force in the absence of ENaC β and γ subunits.

**Figure 3:**
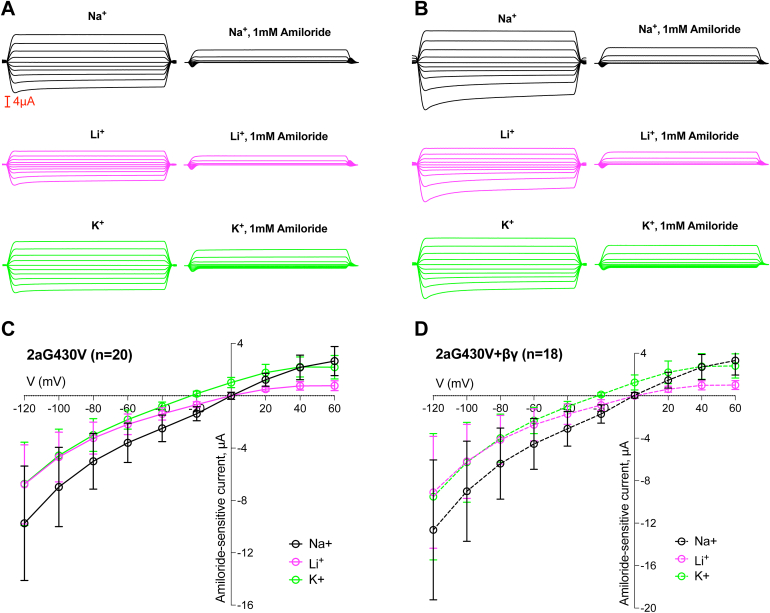
**Ion selectivity of ASIC2aG430V was not altered by ENaC β and γ subunits.** Representative whole-cell recordings of mouse ASIC2aG430V (*A*) or ASIC2aG430V+βγENaC (*B*). Oocytes were voltage-clamped from −140 to +40 mV in 20-mV increments while perfused at a flow rate of 5 ml/min in a buffer containing Na^+^, Li^+^, or K^+^ as the major conducting ion. Amiloride-sensitive currents were determined by subtraction of currents in the presence of 1 mM amiloride from the currents in the absence of amiloride. *C* and *D,* Current-voltage relationship. Amiloride-sensitive currents of Na^+^, Li^+^, or K^+^ were plotted against test potentials in the range of −120 to +40 mV. Data are presented as mean ± SD from 20 oocytes expressing ASIC2aG430V and 18 oocytes expressing ASIC2aG430V+βγENaC.

Since the G430V mutation was introduced within the channel’s pore (45), it might alter the channel’s selectivity to different cations. We measured the whole cell currents in oocytes expressing ASIC2aG430V, or ASIC2aG430V+βγENaC with Na^+^, Li^+^ or K^+^ as the conducting ion in the perfusate. Both ASIC2aG430V and ASICG430V2a+βγENaC are K^+^ permeable with a reverse potential of −20 mV (Fig. 3, *A* and *B*). Also, ASIC2aG430V and ASICG430V2a+βγENaC had lower currents with Li ^+^ as the charge carrier, rather than Na^+^ (Fig. 3, *C* and *D*). In contrast, αβγENaC-mediated Li^+^ currents are greater than Na^+^ currents (46, 47). No difference in ion selectivity was observed between ASIC2aG430V and ASICG430V2a+βγENaC (Fig. 3). Overall, our results suggest that ENaC β and γ subunits had little effect on the cation permeability of ASIC2aG430V channels.

### Contributions of ENaC β and γ subunits to flow-mediated channel activation

**Figure 4:**
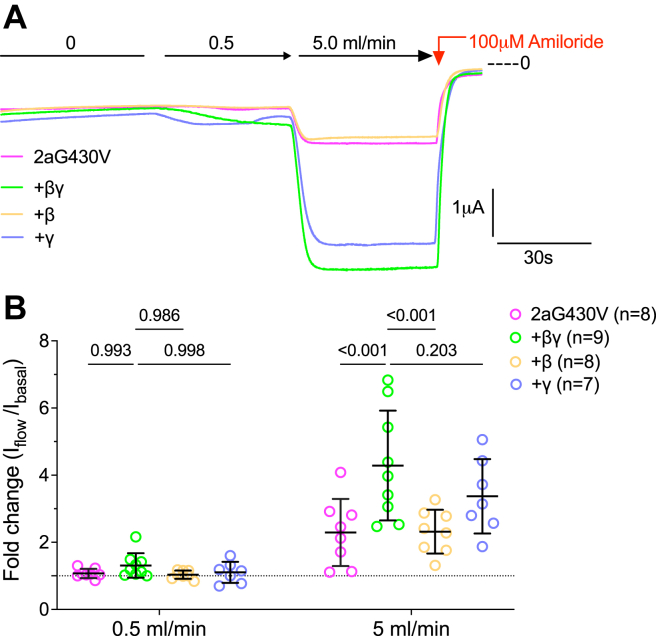
**Subunit contributions to the flow-mediated activation of ASIC2aG430V+βγENaC.***A*, representative current traces of oocytes expressing mouse ASIC2aG430V alone or in the presence of ENaC β, γ or both β and γ subunits. Flow rates were changed from 0 to 0.5 and then to 5 ml/min. 100 μM amiloride was added to the bath at the end of the recording. *B*, summary of the fold change of flow-activated currents (I_flow_/I_basal_) at the two flow rates in oocytes expressing mouse ASIC2aG430V alone (n = 8) or in the presence of ENaC β (n = 8), γ (n = 7) or both β and γ subunits (n = 9). Statistical comparisons were performed with 2-way ANOVA, followed by Sidak’s multiple comparison test.

We observed that a 10-fold increase in perfusion rate (0.5–5 ml/min) led to significant channel activation in oocytes co-expressing ASIC2aG430V with ENaC β and γ subunits (Fig. 2). To identify whether both ENaC subunits are required to confer flow sensitivity, we examined the channel’s response to different flow rates (0 to 0.5 to 5 ml/min) in oocytes expressing ASIC2aG430V alone or co-expressing βENaC, γENaC, or both β and γENaC (Fig. 4*A*). When the perfusion was switched on (0–0.5 ml/min), only negligible changes in whole Na^+^ currents were observed in all four different channel types (Fig. 4). However, the high flow rate (5 ml/min) elicited differential responses among the four groups. There were two-fold increases in Na^+^ currents in oocytes expressing ASIC2aG430V or ASIC2aG430V+βENaC, which was further increased to approximately four-fold when ASIC2aG430V was co-expressed with γENaC or with both β and γ subunits (Fig. 4*B*). Together, our data suggest that the presence of γENaC confers higher sensitivity to flow-mediated channel activation in ASIC2aG430V channels.

**Figure 5:**
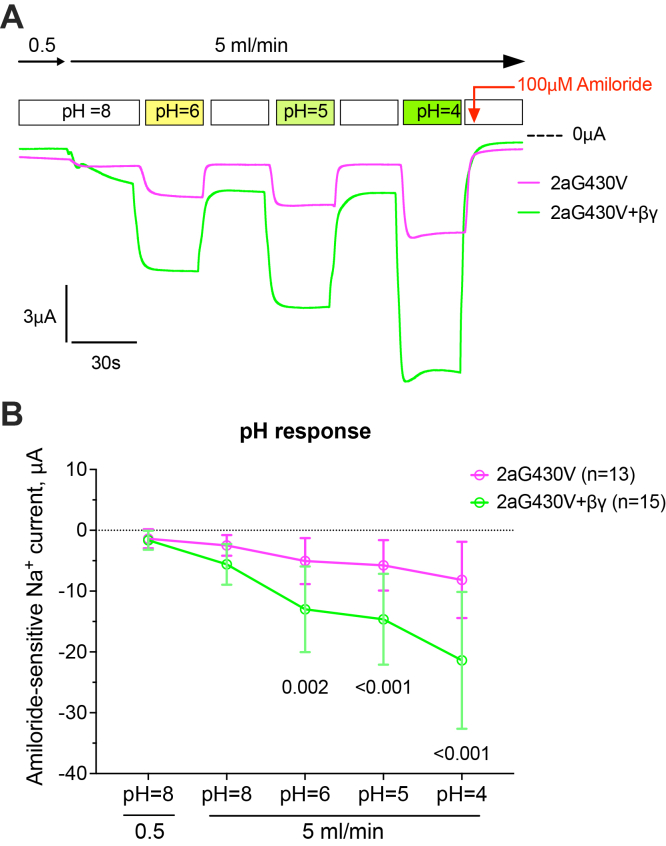
**Proton-mediated ASIC2aG430V activation was enhanced by ENaC β and γ subunits.***A*, representative current traces of oocytes expressing mouse ASIC2aG430V or ASIC2aG430V+βγENaC. The flow rates were changed from 0.5 to 5 ml/min at pH of 8. The channel’s responses to pH changes were tested by switching to acidic perfusion buffer (pH of 6, 5 and 4) from pH = 8 at a flow rate of 5 ml/min. 100 μM amiloride was added to the bath at the end of the recording. *B*, summary of the whole cell Na^+^ currents elicited by acidic pHs. Data are presented as mean ± SD, and the number of oocytes (n) assayed for each group is indicated. Statistical comparisons were performed with 2-way ANOVA, followed by Sidak’s multiple comparison test.

We also examined whether the presence of ENaC β and γ subunits also alters the channel’s response to other gating stimuli. It has been well established that ASIC channels reside in a closed state at neutral pH and are transiently activated by reductions in extracellular pH to a peak current, which gradually declines to the plateau current (desensitized state) upon continuous exposure to low pH (see Fig. 1*A*). However, replacement of Gly 430 with Val (ASIC2aG430V) resulted in sustained channel activation at an acidic pH of 6, 5 or 4 in both ASIC2aG430V and ASIC2aG430V+βγENaC channels (Fig. 5*A*). Also, we detected larger whole cell Na^+^ currents in oocytes expressing ASIC2aG430V+βγENaC, compared to ASIC2aG430V alone, at a pH of 6, 5 or 4 under a high perfusion rate (Fig. 5*B*).

### ASIC2a interacts with ENaC β and γ subunits in HEK293 cells

**Figure 6:**
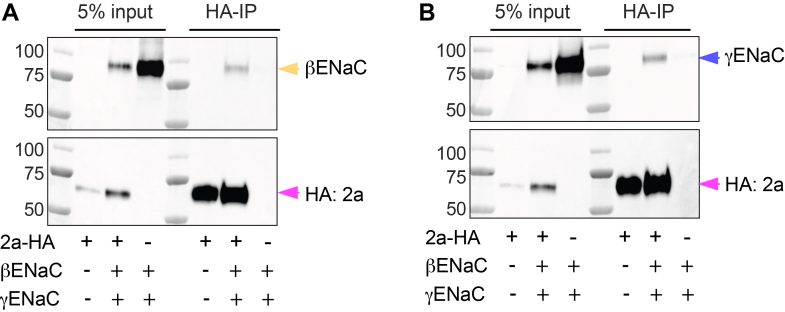
**2aG430V interacts with both ENaC β and γ subunit.** HEK293 cells were transiently co-transfected with a ASIC2aG430V construct that has a C-terminal HA epitope tag (2a-HA), ENaC β and γ subunits (2a-HA+βγENaC). Cells expressing only ASIC2a-HA or βγENaC were included as negative controls of HA beads pulldown. 5% of the whole cell (WC) lysates were saved as WC inputs and the remainder were incubated with ChromoTek HA-Trap Agarose to pull down ASIC2aG430V-HA and its associated proteins. The expression of ENaC β (*A*) or γ subunit (*B*) was probed in the HA-IPs and the WC Input. Successful ASIC2aG430V-HA pulldowns were confirmed by re-probing the blots with HA antibodies (*bottom panels*). Markers of molecular weight (kDa) are shown on the *left*. Experiments were repeated in HEK293 cells of four different passages with separate transfections.

The observation that flow-mediated channel activation of ASIC2aG430V was further augmented only by γENaC, not βENaC (Fig. 4), raises the question whether ASIC2a assembles with both the β and γ subunits to form mechanosensitive channels. We examined this question with a co-immunoprecipitation (co-IP) assay in HEK293 cells co-expressing ASIC2aG430V with βENaC and γENaC. To facilitate immunodetection and pulldown of the ASIC2a, an HA-epitope tag was inserted at the C-terminus of ASIC2aG430V. As shown in Figure 6, ASIC2aG430-HA was detected in the whole cell lysates and successfully isolated with HA-Trap agarose. Both βENaC and γENaC were specifically detected in HA-IPs of cells co-expressing ASIC2aG430-HA, representing approximately 4 to 7% of the whole cell input (Fig. 6). Only negligible signals were detected in HA-IPs of cells transfected only with β and γENaC (<0.2% of the whole cell input), despite having higher whole cell expression of these two proteins (Fig. 6). Furthermore, no β or γENaC signal was detected in HA-IPs of cells transfected only with ASIC2aG430-HA (Fig. 6). Together, our data suggest that both β and γENaC associate with ASIC2a within the channel complex and their interaction is independent of the other ENaC subunit.

### Structural modeling supports assembly of ASIC2a with ENaC β and γ subunits

**Figure 7:**
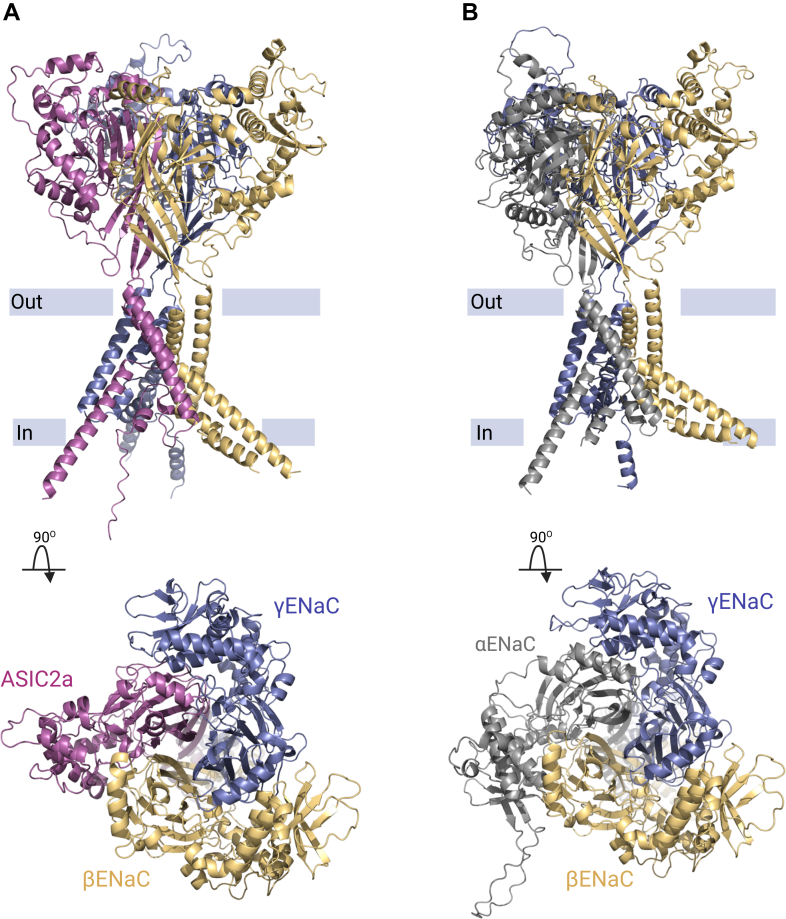
**AlphaFold3 structural modeling supports assembly of ASIC2a with ENaC β and γ subunits.** Ribbon representations of AlphaFold3 models of ASIC2a–βγENaC (*A*) and canonical αβγENaC (*B*), viewed from the side (t*op panels*) or the extracellular face (*bottom panels*). Subunits are colored individually (ASIC2a, *magenta*; αENaC, *gray*; β, golden; γ, *blue*).

To assess whether ASIC2a can substitute for αENaC in the trimeric channel architecture without major steric incompatibility, we used AlphaFold3 to generate structural models of both the canonical αβγENaC trimer and the novel ASIC2a–βγENaC heterotrimer (Fig. 7). No steric clashes were detected at any subunit interface in either model. To quantify interface quality, we used the Protein Interfaces, Surfaces and Assemblies (PISA) server to calculate buried surface area, assembly free energy, hydrogen bonds, and salt bridges at each subunit interface (Table 1). In the αβγENaC model, which recapitulated the cryo-em structure of the extracellular domains (PDB code 6WTH), the three subunit interfaces had buried surface areas consistent with large, well-packed contacts. In the ASIC2a–βγENaC model, the β–γ interface was well preserved with 93% of the buried surface area of the canonical complex, and similar numbers of hydrogen bonds and salt bridges. The ASIC2a–γ interface was similarly large (92% of α–γ), with an assembly free energy of −29.6 kcal/mol and 34 hydrogen bonds. The ASIC2a–β interface, while smaller (70% of α–β), retained 28 hydrogen bonds and 11 salt bridges with a ΔG of −18.8 kcal/mol, consistent with a stable subunit contact. Taken together, structural modeling suggests that ASIC2a can occupy the αENaC position within the trimeric channel complex without a major disruption to subunit packing.

**Table 1:** Subunit interface properties of AlphaFold 3 models of ASIC2a or α ENaC subunits assembled with βγENaC

Interface	BSA (Å^2^)	ΔG (kcal/mol)	N_HB_	N_SB_
αβγENaC				
α – β	3919	−26.6	51	15
α – γ	4189	−26.5	50	29
β – γ	4235	−36.9	56	23
ASIC2a–βγENaC				
ASIC2a – β	2727	−18.8	28	11
ASIC2a – γ	3839	−29.6	34	11
β – γ	3936	−36.9	50	22

BSA, buried surface area; ΔG, assembly free energy; N_HB_, hydrogen bonds; N_SB_, salt bridges.

Values from PISA analysis of AlphaFold3 models of each complex. Steric clashes were not detected at any interface in either model.

## Discussion

Our previous work suggested that ASIC2 and ENaC subunits are key components of a large multimeric protein complex within - vascular SMCs for sensing pressure-induced mechanical stretch and the transduction of it to cellular electrical signals during pressure-induced vasoconstriction (22, 23). In the present study, we examined the mechanosensitivity of a channel, comprised of ASIC2a and ENaC β and γ subunits, by measuring the channel’s response to flow-induced shear force in an oocytes expression system. We found that oocytes expressing ASIC2a had very low baseline currents at a pH of 8 and were insensitive to a 10-fold increase of perfusion rates (Fig. 1). In contrast, amiloride-sensitive inward Na^+^ currents were readily detectable in a gain-of-function mutant, ASIC2aG430V, whose channel activity was enhanced by a high bath perfusion rate (Fig. 2). The co-expression of β and γENaC led to greater mechanosensitivity in oocytes also expressing ASIC2a or the gain-of-function mutant ASIC2aG430V (Figs. 1 and 2), supporting our hypothesis that ASIC2a assembles with ENaC β and γ subunits to form mechanosensitive ion channels. In addition, our model of ASIC2a-β-γ heterotrimer (Fig. 7) suggests that these subunits can assemble without major steric clashes. Future studies of single-channel properties using patch clamp should provide further insights into ASIC2a+βγENaC and ASICG430V2a+βγENaC channel behavior.

It was surprising that flow-mediated activation of ASIC2aG430V was selectively enhanced by γENaC, not βENaC (Fig. 4). Our co-IP results showed that both ENaC β and γ subunits interact with ASIC2a within a channel complex to form mechanosensitive channels (Fig. 6). Canonical ENaCs are comprised of three homologous subunits. With ENaC subunits, it is clear that αENaC can assemble alone to form homotrimeric channels, with either β or γ subunit to form functional αβ or αγ channels, or with β and γ subunits to form αβγ channels in heterologous expression systems (34, 48). Different subunit compositions result in channels with distinct functional properties. For example, αβ channels were more permeable to Na^+^ than Li^+^, and less sensitive to amiloride and related analogs than channels including a γ subunit (49). αβ channels had high channel open probability (P_O_) and reduced inhibition by extracellular Na^+^, suggesting that the γ subunit has roles in restraining ENaC activity (50, 51). Importantly, channel’s response to flow-mediate shear stress is also affected by ENaC subunit compositions. Although the γ subunit dose not form a functional channel by itself, shear stress-induced channel activation is higher in channels containing the γ subunit (αγ or δγ) than the β subunits (αβ or δβ), respectively (52).

Our previous studies examining the functional effects of extracellular mutations on ENaC function suggested that α and γ subunit mutations at specific sites led to significant and large changes in ENaC function, as assessed by changes in the channel’s Na^+^ self-inhibition response. In contrast, β subunit mutations, in general, resulted in no or modest effects on ENaC function (53, 54, 55, 56, 57, 58, 59). Recent structural studies suggest that the β subunit extracellular domain serves as a structural scaffold (32), supporting our observations that ASIC2aG430V require co-expression of γENaC, rather than βENaC, to enhance the sensitivity of the channel to flow. The lack of contribution of βENaC to flow sensitivity was a surprising finding given the importance of βENaC to strain-sensing in isolated renal vascular SMCs and small renal arteries and arterioles (19, 26, 44, 60). This difference may reflect the importance of individual ENaC subunits in modal specificity to activate ASIC2-β/γENaC. We previously identified a number of key sites within the pore-forming region of the γ subunit required for ENaC activation in response to flow-induced shear stress (61), and found a similar role of *C. elegans* MEC-10 in conferring mechanosensitivity to the MEC-4/MEC-10 channel complex (62). Take together, these findings suggest that γENaC harbors important structural features required for a robust response to mechanical force.

Our model of vascular SMCs' mechanosensing suggests that the primary mechanosensor is a heteromultimeric protein complex, where the ion-conducting channel is physically linked with the extracellular matrix and the intracellular cytoskeleton (23). A similar model has also been proposed for ENaC-mediated mechanosensing in vascular endothelial cells (63). New studies from Fronius’s group suggest that ENaC is physically tethered to endothelial glycocalyx through N-glycans attached to the α subunit, and this interaction is important for endothelial cells to sense shear stress and normal vascular function (64, 65). Removal of these asparagine residues in αENaC reduced the channel’s shear stress response, while engineering the corresponding N-glycosylation sites in δENaC enhanced the channel’s response to shear stress (64, 65). Additionally, hyaluronidase treatment to disrupt glycocalyx integrity prevented the effect of amiloride on flow-mediated dilation in carotid arteries isolated from mice (64). The interaction between the mechanosensing channel complex and extracellular matrix/intracellular cytoskeleton may require additional axillary proteins. Stomatin proteins and their nematode orthologues have been implicated to regulate the gating of ENaC/degenerin channels, likely through stabilizing channel’s surface expression and its clustering within the mechanosensing complex (66, 67, 68, 69, 70, 71, 72, 73). Future studies are needed to identify other components of this mechanosensory complex within vascular SMCs.

## Experimental procedures

### cDNA constructs, site-directed mutagenesis and *in vitro* transcription

Mouse ENaC α, β and γ subunits cDNAs were cloned into pBluescript SK(−) from a mouse kidney cDNA library (74). Wild type (WT) mouse ASIC2a in pcDNA3 was a generous gift from Dr Candice Askwith (75). The degenerin site mutation in ASIC2a (ASIC2aG430V) was introduced using the Q5 Site-Directed Mutagenesis Kit (NEB, E0554S) using the WT ASIC2a as a template. Subsequently, a HA epitope tag was inserted at the C-terminus of the ASIC2aG430V construct using the same kit. Standard DNA sequencing was performed to confirm the desired editing. cRNAs of ENaC subunits or ASIC2a constructs were synthesized using Invitrogen mMESSAGE mMACHINE kits (ThermoFisher, AM1344 and AM1348) and purified using RNeasy MinElute Cleanup Kit (Qiagen, 74,204).

### Xenopus Oocyte harvest and micro-injection

Oocytes were harvested from mature female *Xenopus laevis*, defolliculated with collagenase treatment, and maintained at 18 °C in modified Barth's saline (MBS: 88 mM NaCl, 1 mM KCl, 2.4 mM NaHCO_3_, 15 mM HEPES, 0.3 mM Ca(NO_3_)_2_, 0.41 mM CaCl_2_, 0.82 mM MgSO_4_, pH adjusted to 7.4) supplemented with 10 μg/ml sodium penicillin, 10 μg/ml streptomycin sulfate and 100 μg/ml gentamicin sulfate. Stage V-VI oocytes were injected with cRNA mixtures of 2 ng ASIC2a (WT or G430V) with or without an equal amount of ENaC β or γ subunit. In some experiments, a group of oocytes were injected with mouse α, β and γENaC (1 ng/subunit) as the control. Following microinjection, oocytes were maintained at 18 °C in MBS for 24-72 h for optimal channel expression. All chemicals were purchased from Sigma-Aldrich unless stated otherwise. The protocol for harvesting oocytes from *X. laevis* was approved by the University of Pittsburgh’s Institutional Animal Care and Use Committee.

### Electrophysiology studies in oocytes

Two-electrode voltage clamp (TEV) was performed at room temperature using a GeneClamp 500B amplifier (Axon Instruments). Data were acquired with Clampex 10 (Molecular Devices) using a DigiData 1440 interface (Axon Instruments). Recording pipettes were filled with 3 M KCl with resistances of 0.3 to 3 MΩ. Oocytes were placed in a recording chamber (Automate, OPC-1-110) that was constantly perfused with the NaCl-110 solution (110 mM NaCl, 2 mM KCl, 1.54 mM CaCl_2_, and 10 mM HEPES, pH adjusted to 8 with NaOH) at a rate of 0.5 ml/min. Oocytes were clamped at −100 mV while whole cell Na^+^ currents were continuously recorded. To assess the effect of flow-induced shear stress (FSS) on channel activity, the flow rate was increased to 5 ml/min until currents reached a plateau (30–60s). At the end of each recording, amiloride (10 or 100 μM) was added to bath perfusion to block the channel and determine leak currents. Three measurements were taken: (i) the current measured at the low flow rate (0.5 ml/min, *I*_*basal*_), (ii) the plateau current measured at the high flow rate (5 ml/min, *I*_*flow*_), (iii) and the current measured at the high flow rate after the application of amiloride.

To examine the response of the channel to protons, oocytes were exposed to NaCl-110 buffers with increasing acidity (pH = 8 to pH = 6, pH = 5 or pH = 4) at high flow rate while whole cell currents were continuously recorded. Acidic buffers were prepared with 10 mM MES instead of HEPES. For ion selectivity analysis, bath solutions containing Na^+^, Li^+^, or K^+^ as the major conducting cation were prepared by replacing NaCl in the NaCl-110 solution with equal molar of LiCl or KCl. The pH was adjusted to 8 with NaOH, LiOH, or KOH, respectively. Oocytes were perfused for 1 min with each solution, and whole cell currents were determined at varying holding potentials (−140–60 mV in 20-mV steps, 0.5 s/step). Oocytes were then perfused for 20 s with each solution in the presence of 1 mM amiloride, after which whole cell currents were measured at the different holding potentials.

To determine the channel’s sensitivity to amiloride, whole cell Na^+^ currents were continuously recorded at the perfusion rate of 5 ml/min while exposing to increasing concentrations of amiloride (10^−7^, 10^−6^, 10^−5^, 10^−4^ and 10^−3^ M). Oocytes were perfused with each concentration of amiloride for 30 s to obtain stabilized currents. Normalized whole cell currents were plotted as a function of amiloride concentrations (M), and dose-response curves were generated by fitting data to the following sigmoidal equation: Y = 1/(1 + 10^(logIC50 − logX)^) where X is the concentration of benzamil, and IC_50_ is defined as the concentration of amiloride that inhibits 50% of the whole cell Na^+^ current. Comparisons between the dose-response curves were analyzed with the extra sum-of-squares F test.

### Co-immunoprecipitation assay

HEK293 cells were transiently transfected with plasmids encoding mouse ASIC2aG430V-HA, ENaC β and γ subunit, or ASIC2aG430V-HA+βγ using Lipofectamine 3000 (Invitrogen). The next day, cells were extracted with in detergent solution (20 mm HEPES, 100 mm NaCl, 40 mm KCl, 1 mm EDTA, 10% glycerol, 1% Nonidet P-40, 0.4% deoxycholate, pH = 7.4) supplemented with protease inhibitor mixture III (Calbiochem). Insoluble material was removed by centrifugation at 14,000 rpm for 7 min at 4 °C. 5% aliquots of the supernatant were retained as whole cell inputs, while the remainder was incubated with ChromoTek HA-Trap agarose (ProteinTech, #ata) for 1.5 h at 4 °C with end-over-end mixing. The agarose beads were washed three times with a HEPES-buffered saline (10 mm HEPES, 150 mm NaCl with phosphatase inhibitors, pH = 7.4) before being eluted into 2X Laemmli sample buffer (Bio-Rad, #1610737). Both the whole cell inputs and HA agarose elution (HA-IPs) were separated on a 4 to 15% Criterion gel (Bio-Rad, # 56780840) and transferred to nitrocellulose membrane (Millipore, HATF00010). The presence of ENaC subunits in the whole-cell lysate and HA pulldowns was detected using rabbit anti-βENaC antibody (1:1000, a generous gift from Dr Lawrence Palmer) or with anti-γENaC antibody (1 μg/ml; Stressmarq, SPC405D), followed by an incubation with HRP-conjugated anti-rabbit IgG secondary antibodies (Jackson ImmunoResearch, #711-035-152). Successful HA pulldown was confirmed by reblotting the membranes with HRP-conjugated anti-HA antibodies (1:1000; Roche, 3F10). Chemiluminescence signals were detected using Clarity Western ECL Substrate (Bio-Rad, #1705061) and imaged with the Bio-Rad ChemiDoc system. Band densities were quantified using Image Lab, a Bio-Rad software.

### Data and statistical analyses

Experiments were repeated with a minimum of two batches of oocytes obtained from different frogs. Electrophysiology data were analyzed using Clampfit10 (Molecular Devices). Data were expressed as the mean ± standard deviation (SD). Prism (GraphPad Software) was used for graphing and statistical analysis. Scatter-dot plots were used to show data points along with horizontal bars for the mean and SD. D'Agostino-Pearson normality test or QQ plot was performed to assess data distribution. Statistical comparisons were obtained from an unpaired Student *t* test, or from analysis of variance (ANOVA) followed by appropriate *post hoc* tests. A *p* value of less than 0.05 was considered statistically different.

### Structural modeling and interface analysis

Structural models of mouse αβγENaC and ASIC2a–βγENaC heterotrimers were generated using the AlphaFold3 server (alphafoldserver.com) with full-length subunit sequences as inputs. Five independent models were generated for each complex; the highest-ranked model (by AlphaFold3 ranking score) was selected for interface analysis. Disordered intracellular termini from each subunit were trimmed prior to submission to the Protein Interfaces, Surfaces and Assemblies (PISA) server (https://www.ebi.ac.uk/pdbe/pisa/) for analysis of buried surface area, assembly free energy (ΔG), hydrogen bonds, and salt bridges at each pairwise subunit interface. Buried surface area was calculated as the solvent-accessible surface area lost upon subunit association.

## Data availability

All electrophysiological recordings presented in the manuscript have been archived and are fully available to the journal upon reasonable request.

## Conflict of interest

The authors declare that they have no conflicts of interest with the contents of this article.
